# Transportation noise and annoyance related to road traffic in the French RECORD study

**DOI:** 10.1186/1476-072X-12-44

**Published:** 2013-10-02

**Authors:** Julie Méline, Andraea Van Hulst, Frédérique Thomas, Noëlla Karusisi, Basile Chaix

**Affiliations:** 1 Inserm, Paris, U707, France; 2 Université Pierre et Marie Curie-Paris6, UMR-S 707, Faculté de Médecine Saint-Antoine, Paris, France; 3 Department of Social and Preventive Medicine, Faculty of Medicine, University of Montreal, Montreal, Canada; 4 Research Center of Sainte-Justine, University Hospital, Montreal, Canada; 5 Centre d’Investigations Préventives et Cliniques, Paris, France

**Keywords:** Transportation noise, Annoyance, Residential neighborhood

## Abstract

Road traffic and related noise is a major source of annoyance and impairment to health in urban areas. Many areas exposed to road traffic noise are also exposed to rail and air traffic noise. The resulting annoyance may depend on individual/neighborhood socio-demographic factors. Nevertheless, few studies have taken into account the confounding or modifying factors in the relationship between transportation noise and annoyance due to road traffic. In this study, we address these issues by combining Geographic Information Systems and epidemiologic methods. Street network buffers with a radius of 500 m were defined around the place of residence of the 7290 participants of the RECORD Cohort in Ile-de-France. Estimated outdoor traffic noise levels (road, rail, and air separately) were assessed at each place of residence and in each of these buffers. Higher levels of exposure to noise were documented in low educated neighborhoods. Multilevel logistic regression models documented positive associations between road traffic noise and annoyance due to road traffic, after adjusting for individual/neighborhood socioeconomic conditions. There was no evidence that the association was of different magnitude when noise was measured at the place of residence or in the residential neighborhood. However, the strength of the association between neighborhood noise exposure and annoyance increased when considering a higher percentile in the distribution of noise in each neighborhood. Road traffic noise estimated at the place of residence and road traffic noise in the residential neighborhood (75th percentile) were independently associated with annoyance, when adjusted for each other. Interactions of effects indicated that the relationship between road traffic noise exposure in the residential neighborhood and annoyance was stronger in affluent and high educated neighborhoods. Overall, our findings suggest that it is useful to take into account (i) the exposure to transportation noise in the residential neighborhood rather than only at the residence, (ii) different percentiles of noise exposure in the residential neighborhood, and (iii) the socioeconomic characteristics of the residential neighborhood to explain variations in annoyance due to road traffic in the neighborhood.

## Introduction

Road transportation is the first means of transport in urban areas and is one of the main sources of impairment of residential quality and discourages recreation in residential environments. Among all nuisances, noise is the first source of annoyance mentioned by the Ile-de-France residents [1]. This major annoyance related to transportation noise could lead to deleterious effects on health such as sleeping disorders [2,3], stress, and risk of cardiovascular diseases [2,4]. Annoyance due to transportation noise is well documented [5] by studies and meta-analyses that assessed levels of annoyance for each level of traffic noise [2,3,5-13]. Similarly, according to Bruitparif [14], road, rail, and air traffic are respectively the first, the third, and the second sources of annoyance in the Ile-de-France region (the Paris region).

Many areas exposed to road traffic noise are also exposed to aircraft and railway noise [9]. However, most studies have taken into account the emissions of a unique transportation mode (road, rail, or air traffic) when documenting associations between noise and annoyance [15,16]. Moreover, in addition to the source of noise, the degree of annoyance may also depend on socio-demographic factors including gender, age, education and income levels [5,9]. However, only few studies investigating the relationships between transportation noise and annoyance have either adjusted for individual and specifically neighborhood socio-demographic factors or have considered these variables as potential modifiers of the association of interest [17,18].

In this study, we examined the associations between outdoor road, rail, and air traffic noise and annoyance related to road traffic in the Ile-de-France region, after adjusting for individual and neighborhood socioeconomic factors and for a typology of neighborhoods based on multiple urban and environmental factors. We compared the relationships that were documented between transportation noise and annoyance due to road traffic: 1) when different objective sources of transportation noise were considered (i.e., road traffic, rail traffic and air traffic) and 2) when exposure to outdoor transportation noise was assessed at the exact place of residence or in the neighborhood around the residence. Rail traffic noise was taken into account based on the hypothesis that the addition of other noise nuisances to the road traffic noise could strengthen the feeling that there is too much road traffic in the neighborhood (synergistic effect of different sources of noise). Moreover, in addition to an analysis of the spatial distribution of exposure to transportation noise in the Ile-de-France region, we examined whether the associations between transportation noise and annoyance due to road traffic were modified by individual and neighborhood socio-economic factors.

## Materials and methods

### Study population

The Residential Environment and CORonary heart Disease (RECORD) Cohort (http://www.record-study.org) comprises 7290 residents of the Ile-de-France region who were recruited between March 2007 and February 2008. The participants were recruited during 2-hour medical checkups conducted in four health centers affiliated with the Centre d’Investigations Préventives et Cliniques located in the Ile-de-France region. As eligibility criteria, participants were 30-79 years old, were able to fill survey questionnaires in French, and had to reside in one of 10 (out of 20) administrative divisions of Paris or in 111 other municipalities selected in the Paris metropolitan area. The *a priori* selection of these municipalities aimed to include suburban and urban areas from contrasted socioeconomic backgrounds. All participants (100%) were precisely geocoded based on their residential address in 2007-2008. Additional details on the study are reported elsewhere [19]. The study protocol was approved by the French Data Protection Authority.

### Annoyance due to road traffic

In a study of the concept of noise annoyance conducted by a panel of experts of different countries in charge of evaluating noise annoyance [20], noise annoyance was closely associated with the notion of “nuisance” and “disturbance”. Annoyance was also linked to the concepts of negative feelings and evaluations [18,20]. Annoyance related to road traffic was defined from the RECORD Study questionnaire based on the following item: “do you find that in your neighborhood there is too much road traffic?”. This item put the emphasis on a negative aspect (“too much”) and was therefore referring to “road traffic nuisance” or in other terms to annoyance due to road traffic (thus a broader concept than “road traffic noise annoyance” itself since the survey question was related to all aspects of road traffic nuisance indistinctively: noise, air pollution, security, etc.). The degree of annoyance was rated on a 4 level scale: 'Yes, definitely’, 'Yes, probably’, 'Probably not’, and 'Definitely not’. A binary variable of annoyance was defined with value 1 for the 'Yes, definitely’ answer, and 0 otherwise.

### Individual and neighborhood variables

Gender was coded as a binary variable. Age was categorized into 3 classes: 30-44; 45-59; 60-79 years old. Education was divided into 4 classes: no education (low); primary and lower secondary education (middle-low); higher secondary and lower tertiary education (middle-high); and upper tertiary education (high). Nonownership of dwelling was coded as a binary variable.

Based on separate sources of data geocoded at the building level, two neighborhood socioeconomic variables were defined in buffers of 500 m of radius centered on the residence of the participants. These buffers took into account the street and road network around the residence (i.e., the radius of 500 m was defined along the street network). The educational level of residents in the neighborhood was defined as the proportion of residents aged >25 years with an upper tertiary education (2006 Census). The median income in 2006 (General Directorate of Taxation) of households residing in these buffers was also determined. These two variables were then divided into 4 categories with a similar number of participants. We also distinguished the participants residing in the city of Paris (county #75), the participants living in the “inner suburbs” (first belt of counties around Paris; counties #92, 93, and 94), and the participants living in the “outer suburbs” (second belt of counties around Paris; counties #78, 91, 94, and 95).

Finally, we used a typology of neighborhoods elaborated for the RECORD Study in the Ile-de-France region [21]. This typology, established in two steps with a factor analysis and a cluster analysis, provides a grouping of neighborhoods with comparable characteristics but which are not necessarily geographically adjacent. Six neighborhood types were identified from the combination of 13 neighborhood variables (among the numerous variables initially considered), including: 4 indicators of the built environment (proportion of the neighborhood area covered with buildings, density of intersections, average street block length, deterioration of the physical environment in the neighborhood); 2 indicators of air pollution (measured concentrations of PM_10_ and NO_2_ in the neighborhood); 4 indicators of the service environment (total number of destinations, number of supermarkets, number of grocery stores, incoming and outgoing traffic by public transportation); and 3 indicators of neighborhood social interactions (neighborhood stressful social interactions, neighborhood mistrust and hostility, and stigmatized neighborhood identity). The detailed methodology to derive this neighborhood typology has been reported elsewhere [21]. Two urban central neighborhoods, two urban neighborhoods, and two suburban neighborhoods were identified, with more or less advantaged social interactions in each urbanization stratum.

### Transportation noise variables

The (road, rail, air) transportation noise data were provided by Bruitparif. This non-Governmental Organization is in charge of gathering published layers of noise modeled by each municipality or grouping of municipalities in the Paris metropolitan area from 2007 onwards, according to the Environmental Noise Directive [2]. We chose to use these noise maps to characterize transportation noise exposure at the place of residence and residential neighborhood scales and to analyze the relationship with annoyance due to road traffic reported for the residential neighborhood because a high correspondence has been documented between the relationships of observed or predicted noise exposure with noise annoyance at these scales [22]. The measurement of noise in this database is in dB(A) and is expressed with the standard European Lden and Ln indicators. In the dB(A) unit of measurement (Decibel with a A-weighted filter), the filter A scale corresponds to people’s natural hearing sensitivity recognition at different sound frequencies [5,9]. Following previous work [23], we chose to use the Lden indicator, defined as the A-weighted equivalent continuous noise level (LAeq) over a 24 h period but in which levels during the evening and night are increased by 5 dB(A) and 10 dB(A), respectively.

The modeled layers for each municipality were obtained by Bruitparif from two types of institutions: local authorities and government services. According to the Environmental Noise Directive, the local authorities had to elaborate complete maps of road, rail, and air traffic noise. As the local authorities relied on different engineering offices to estimate noise levels on their territory, there was some heterogeneity in the noise modeling methodology. For instance in Paris, the commissioned engineering office used the EASYMAP model (SIRIATECH, Roubaix, France). This model was based on (1) the environmental noise prediction software MITHRA (Scientific and Technical Centre for Buildings, Grenoble, France), (2) the geographical information system ArcGIS (ESRI, Redlands, California, USA) and (3) the environmental management information system Drag&Fly (SIRIATECH, Roubaix, France) to generate noise calculations and noise mapping across Paris in two or three dimensions. Additional explanations (input parameters, detailed methodology) on the modeling of noise in Paris are provided in a previous article [23].

The layer provided by the city of Paris was built from a raster with noise information on a 2 × 2 meter cell grid, at 1.5 meter from the ground, and taking into account the distribution of buildings. Differently, the layers provided by the other cities in the Ile-de-France region were vector files of noise points or noise lines, modeled every 2 meters at 4 meters from the ground and at 2 meters from the buildings’ frontage. Despite this heterogeneity, an overall noise map was built by Bruitparif to comply with the Environmental Noise Directive Recommendations. When municipalities did not generate or release a noise database for their territory, Bruitparif completed the missing information with a noise database elaborated by government services. These institutions had to elaborate maps of road, rail, and air traffic noise that were less precise than those produced by the local authorities. Indeed, only roads with more than 6 million vehicles per year, railways with more than 60 000 trains per year, and airports with more than 50 000 movements per year were taken into account in this governmental modeling of noise. Bruitparif processed and homogenized all these layers according to the Environmental Noise Directive, in order to create a noise database at the scale of the Ile-de-France region. From the vector layers of noise points or noise lines and the raster layer provided by local authorities and government services with noise levels between 30 and 80 dB(A), Bruitparif generated a layer of polygons of noise levels with a subdivision in 5 to 5 dB(A) classes (55 – <60; 60 – <65; 65 – <70; 70 – <75; and 75- < 80). The raw data of the final Bruitparif map were collected between 2007 and 2011.

The geographical processing of the noise database was performed with the ArcInfo 10 Geographic Information System. The Environmental Noise Directive established that noise levels equal to or above 55 dB(A) could have an impact on human health. However, in order to take into account the heterogeneous environments in the Ile-de-France region, from quiet rural areas to busy urban areas, we also integrated levels of noise from the minimum level of 30 dB(A) corresponding to the rural environment at night to the established European level of 55 dB(A). The Bruitparif layers of noise polygons (from 55 to 80 dB(A)) were juxtaposed in order to build one layer of noise polygons for each transportation type (road, rail, air) at the scale of the Ile-de-France region. To integrate all noise classes from 30 to 80 dB(A), we processed and homogenized the original layers of noise points elaborated by local authorities and government services (that include noise classes between 30 and 50 dB(A)) following the general process used by Bruitparif, in order to elaborate layers of noise polygons. The final layer of noise polygons for each transportation mode (road, rail, air) at the scale of the Ile-de-France region was generated by filling missing portions of the Bruitparif layer with information from the layer of polygons from the Government/local authorities (ArcGIS update Tool).

**Figure 1:**
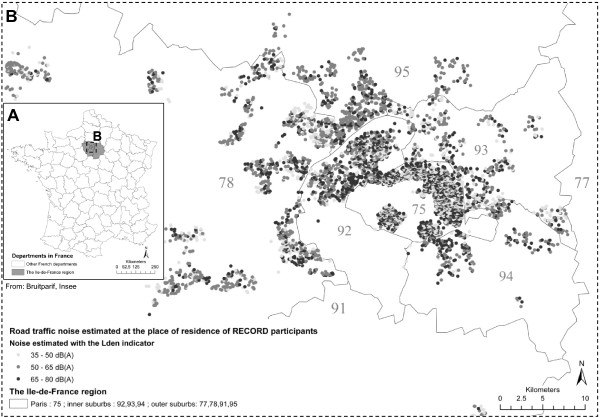
**Location of places of residence of RECORD participants in the Ile-de-France region in France. (A,B)** and distribution of road traffic noise estimated at the place of residence **(B).**

In order to estimate noise exposure at the place of residence of the participants, outdoor road, rail, and air traffic noise levels were extracted at each geocoded place of residence. In order to estimate noise exposure of participants in the residential neighborhood, we determined buffers around the places of residence (Figure 1). These buffers were centered on the exact residential building of the participants and had a radius of 500 m. The shape of the buffers took into account the street and road network. A radius of 500 m was chosen and the street network was taken into consideration in order to characterize as precisely as possible the outdoor noise exposure of participants moving around their residence during the day. Indeed, in most places in the Ile-de-France region, people are likely to find basic services within a 500 m radius around their residence [24]. Moreover, different studies based on the RECORD Study have shown that contextual variables are particularly strongly associated with health outcomes when measured within 500 m radius buffers [25,26].

**Figure 2:**
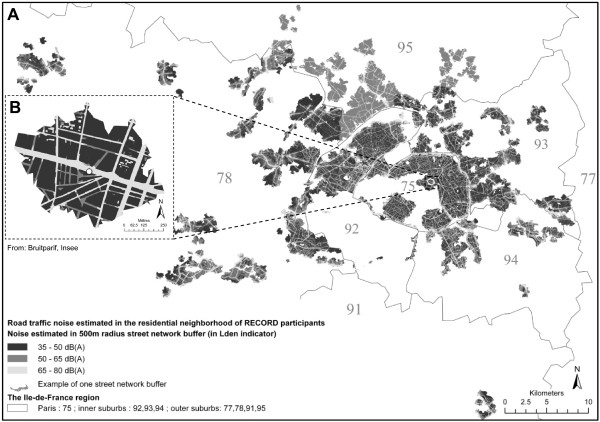
Distribution of road traffic noise estimated in the residential neighborhood for all RECORD participants (A) and for one participant (B) in the Ile-de-France region.

As shown in Figure 2, the ArcGIS intersect tool was used to identify the portion of the different polygons of noise that fell into the buffer of each participant (the operation was performed for each layer of road, rail, and air traffic noise). Then according to the proportion of the surface of each class of noise that fell into the buffer of each participant, the following noise variables were defined with the SAS software: 25th percentile of noise in the buffer of each participant, median noise value, and 75th percentile of the noise value in the buffer of each participant. The median was used rather than the average, because it was found relevant to consider different percentiles in order to take into account the variability of noise exposure into the buffer for people going through it and to identify people exposed to high levels of noise that would not be captured by an indicator of central tendency.

The areas with modeled information on noise did not cover the entire territory of the Ile-de-France region and the areas with modeled information on noise differed in their coverage according to the type of transportation mode. Some counties and some municipalities within certain counties were completely excluded from the modeling and referred to non-modeled administrative territories. These non-modeled administrative territories differed depending on the type of transportation noise (road, rail or air). Within the administrative territories that were part of the modeling, the modeling for a certain source of noise was not performed in parts of the territory that were too far from this source of noise for being affected. Indeed, the accuracy of the distance of noise from the roads or railways from modeling process is defined essentially in taking into account the characteristics of the environmental context (urban or rural) and of the degree of absorption of noise by the environment (ground and building). All these areas were excluded from the analyses because of an absence of modeled data. Therefore, after excluding the missing values and in taking into account the administrative territories that were part of the modeling with modeled noise data (from 30 to 80 dB(A)) defined for each transportation type (road, rail and air), different samples were defined: 6194 and 6539 participants for road traffic noise estimated at the place of residence and in 500 m radius street network buffers, and 3945 and 4265 participants for rail traffic noise estimated at the place of residence and in the buffers. The number of participants who were exposed to aircraft noise in our sample (n = 78 at the place of residence and n = 152 in the residential neighborhood) was too weak to investigate associations between aircraft noise and annoyance, because of a lack of statistical power.

### Statistical analysis

Descriptive analyses assessed variations of neighborhood factors (education, income) according to administrative division (Additional file 1: Table S1) and variations in transportation noise according to the different contextual variables (Table 1 and Additional file 1: Table S2). Multilevel logistic regression analyses were conducted to examine associations between outdoor road and rail traffic noise levels and annoyance related to road traffic. Analyses were based on the different samples excluding observations with missing information for each noise exposure variable: road and rail traffic noise; estimated at the residence or in the residential neighborhood. The multilevel models were estimated with participants nested within census block group neighborhoods. The 1760 census block group neighborhoods represented in the present analysis, defined for the Population Census, are relatively homogeneous in term of sociodemographic and housing characteristics (the median number of residents per neighborhood represented in our study was 2529 in 2006 (interquartile range: 2159 to 3111).

**Table 1:** Spatial distribution of road traffic noise, according to the administrative division in counties, neighborhood urban typology, and neighborhood education (RECORD Cohort study)

**Variables**	**Road traffic noise at the place of residence (with the Lden indicator and in dB(A))**		**Road traffic noise at the 25**th **percentile of 500 m radius street network buffers around the place of residence (with the Lden indicator and in dB(A))**		**Road traffic noise at the median of 500 m radius street network buffers around the place of residence (with the Lden indicator and in dB(A))**		**Road traffic noise at the 75**th **percentile of 500 m radius street network buffers around the place of residence (with the Lden indicator) in dB(A)**	
	**N = 6194**		**N = 6539**		**N = 6539**		**N = 6539**	
	***Mean***	***±SD***	***Mean***	***±SD***	***Mean***	***±SD***	***Mean***	**±SD**
Total	56.31	±11.20	43.97	±7.71	49.58	±9.59	61.04	±6.47
Ile-de-France region								
Outer suburbs	55.70	10.55	44.69	10.27	51.76	9.65	58.41	7.70
Inner suburbs	58.18	9.19	46.89	6.85	53.49	8.29	61.75	6.19
Paris	55.22	13.13	40.13	1.37	43.19	7.32	62.93	4.10
P For Trend*		3.42* 0.0003		-18.98* <.0001		-31.05* <.0001		22.59* <.0001
Neighborhood proportion of highly educated residents								
Low	57.45	±9.13	47.33	±8.07	53.86	±7.35	60.36	±5.45
Mid-low	56.28	±11.11	44.31	±8.51	50.64	±9.86	60.62	±7.47
Mid-high	56.04	±11.81	42.63	±6.91	47.43	±9.71	61.32	±6.31
High	55.29	±12.66	41.22	±5.36	45.83	±9.28	61.94	±6.42
P For Trend*		-1.42* 0.078		-23.56* <.0001		-26.76* <.0001		11.27* <.0001
Neighborhood typology								
Type 1: suburban	55.01	±9.28	47.10	±9.82	54.86	±6.50	60.33	±4.97
Type 2: suburban	55.92	±10.77	43.62	±9.40	50.73	±9.59	57.54	±8.46
Type 3: urban	58.12	±9.38	47.32	±7.33	53.86	±7.73	61.22	±5.50
Type 4: urban	57.57	±10.41	45.46	±7.64	53.24	±9.72	62.71	±6.45
Type 5: central urban	55.71	±12.80	40.00	±0.00	41.54	±4.79	62.26	±3.24
Type 6: central urban	54.63	±13.21	40.79	±3.70	42.39	±6.46	62.73	±4.28
P For Trend**		81.63** <.0001		1831.84** <.0001		963.46** <.0001		562.47** <.0001

* P Values for trend were estimated from the Jonckheere-Terpstra test. ** P Values for trend were estimated from the Kruskall-Wallis test. All neighborhood variables were expressed as ordinal variables. Means and standard deviations were calculated, after excluding individuals with missing values for traffic noise and neighborhood variables. In the Ile-de-France region, “Paris” is the district 75; “inner suburbs” and “outer suburbs” gather respectively districts 92, 93, and 94 and districts 77, 78, 91, and 95. Abbreviations: “Type 1: suburban”: “Type 1: suburban, low social standing”; “Type 2: suburban”: “Type 2: suburban, high social standing”; “Type 3: urban”: “Type 3: urban, low social standing”; “ Type 4: urban”: “Type 4: urban, high social standing”; “Type 5: central urban”: “Type 5: central urban, high social standing”; “Type 6 : central urban”: “Type 6: central urban, intermediate social standing”.

First, for each sample, an empty model was estimated. Second, we derived parsimonious models retaining only the individual/contextual sociodemographic variables that were independently associated with annoyance related to road traffic (among the following variables: gender, age, nonownership of dwelling, individual education, household income, neighborhood median income, neighborhood education, and the neighborhood urban typology) (Model 1 in Table 2). Third, in order to compare associations with noise variables estimated at the place of residence and noise variables estimated in 500 m radius street network buffers, we defined two sets of noise variables: 1) two categorized noise variables (estimated at the place of residence and corresponding to the median noise value in the neighborhood buffer) that were subdivided into classes with cutoff values every 10 dB(A) from 30 to 80 dB(A) and 2) four standardized continuous noise variables, estimated at the place of residence and corresponding to the 25th, 50th, and 75th percentiles of noise values in each neighborhood buffer. The associations of annoyance due to road traffic (adjusted for the individual/neighborhood factors) with the two categorized noise variables correspond to models 2A and 2B in Table 3, and to the models shown in Additional file 1: Table S3, while the associations with the standardized noise variables correspond to models 4A, 4B, 4C and 4D in Table 4 and to the models shown in Additional file 1: Table S4. Fourth, modification of the relationship between road traffic noise and annoyance due to road traffic by rail traffic noise and modification of the relationship between transportation noise and annoyance due to road traffic by individual and neighborhood income and education were tested, both on a multiplicative scale and on an additive scale as previously recommended [27] (Table 5 and Additional file 1: Tables S5 and S6). We did not estimate relationships between outdoor air traffic noise and annoyance due to road traffic because of a lack of statistical power. All regression analyses were conducted with SAS software.

**Table 2:** Associations estimated from multilevel logistic regression between individual/neighborhood socio-demographic factors and annoyance due to road traffic (Model 1) (RECORD cohort study; N = 6539)

**Individual/neighborhood Variables**	**Model 1: Annoyance due to road traffic**	
	**N = 6539**	
	**OR**	**95% CI**
Male (vs Female)	0.96	(0.85 ; 1.10)
Age	1.00	(0.99 ; 1.00)
Nonownership of dwelling (vs Owner)	1.29	(1.12 ; 1.48)
Individual education (vs High)		
Middle-High	1.11	(0.94 ; 1.30)
Mid-low	1.31	(1.10 ; 1.56)
Low	1.28	(1.01 ; 1.64)
Household income (vs High)		
Middle-High	0.99	(0.82 ; 1.21)
Mid-low	1.33	(1.11 ; 1.61)
Low	1.63	(1.33 ; 1.99)
Neighborhood median income in 500 m street network buffers around the place of residence (vs High)		
Middle-high	1.14	(0.91 ; 1.42)
Mid-low	1.33	(1.06 ; 1.67)
Low	1.44	(1.12 ; 1.86)
Neighborhood type (vs Type 2: suburban, high social standing)		
Type 1: suburban, low social standing	1.26	(0.91 ; 1.76)
Type 3: urban, low social standing	1.84	(1.41 ; 2.39)
Type 4: urban, high social standing	1.67	(1.32 ; 2.12)
Type 5: central urban, high social standing	1.72	(1.30 ; 2.28)
Type 6: central urban, intermediate social standing	3.68	(2.90 ; 4.67)
*Between-neighborhood variance*		*0.79 (0.76 ; 0.82)*

A multilevel logistic regression model was estimated after excluding individuals with missing values for road traffic noise variables. This model 1 is the basic model estimated between individual/neighborhood variables and annoyance due to road traffic. The comparable models estimated in the other samples of smaller size yielded similar results and are not shown in Tables.

**Table 3:** Associations estimated from multilevel logistic regression between road traffic noise estimated at the place of residence (2A) and at the median noise value of 500 m radius street network buffers around the place of residence (2B) and annoyance due to road traffic, adjusted for individual/neighborhood socio-demographic factors (RECORD Cohort Study)

		**Model 2A**	**Model 2B**	
		**N = 6194**	**N = 6539**	
	**OR**	**95% CI**	**OR**	**95% CI**
Road traffic noise estimated 2A: at the place of residence; 2B: in the residential neighborhood				
(Lden indicator)				
(vs [ 30 – 40 dB(A) [ )				
[ 40 – 50 dB(A) [	1.15	(0.80 ; 1.65)	1.35	(0.93 ; 1.95)
[ 50 – 60 dB(A) [	0.76	(0.53 ; 1.08)	1.38	(0.98 ; 1.94)
[ 60 – 70 dB(A) [	0.86	(0.61 ; 1.21)	1.80	(1.26 ; 2.56)
[ 70 – 80 dB(A) [	2.26	(1.58 ; 3.21)	3.07	(1.80 ; 5.25)
*Between-neighborhood variance*	*0.78 (0.75 ; 0.81)*		*0.79 (0.76 ; 0.82)*	

Multilevel logistic regression models were estimated after excluding individuals with missing values for road traffic noise variables. These models were estimated between categorical noise variables and annoyance due to road traffic, adjusted for individual/neighborhood factors of basic model 1 (Table 2). Road traffic noise were estimated at the place of residence in Models 2A (N = 6194) and as the median value of 500 m radius street network buffers around the place of residence in model 2B (N = 6539).

**Table 4:** **Associations estimated from multilevel logistic regression between road traffic noise estimated at the place of residence (4A) and at the 25**th **(4B), 50**th **(4C), and 75**th **percentiles (4D) of noise values of 500 m radius street network buffers around the place of residence and annoyance due to road traffic, adjusted for individual/neighborhood socio-demographic factors (RECORD Cohort Study)**

**Road traffic noise at the place of residence**	**Road traffic noise in the residential neighborhood (25th percentile of noise values of buffers)**	**Road traffic noise in the residential neighborhood (median of noise values of buffers)**	**Road traffic noise in residential the neighborhood (75th percentile of noise values of buffers)**
**OR (95% CI)**	**OR (95% CI)**	**OR (95% CI)**	**OR (95% CI)**
Model 4A (N = 6194)	Model 4B (N = 6539)	Model 4C (N = 6539)	Model 4D (N = 6539)
1.20 (1.12 ; 1.28)	1.07 (0.99 ; 1.15)	1.21 (1.11 ; 1.31)	1.29 (1.19 ; 1.40)
*B-N variance: 0.79 (0.76;0.82) Akaike: 29782.3*	*B-N variance: 0.79 (0.76;0.82) Akaike: 31336.4*	*B-N variance: 0.79 (0.76;0.82) Akaike: 31379.9*	*B-N variance: 0.80 (0.77;0.83) Akaike: 31440.9*

Multilevel logistic regression models were estimated after excluding individuals with missing values for road traffic noise variables. These models were estimated between standardized continuous noise variables and annoyance due to road traffic, adjusted for individual/neighborhood factors of basic model 1 (Table 2). Road traffic noise was estimated in 500 m radius street network buffers around the place of residence. Abbreviation: B-N: between-neighborhood.

**Table 5:** Modification of the association between road traffic noise and annoyance due to road traffic, by neighborhood income and education, on the multiplicative scale (RECORD Cohort Study)

	**Neighborhood proportion of highly educated residents**	**Neighborhood median income**
	**β - 95% CI**	**β - 95% CI**
At the place of residence (N = 6194)		
Neighborhood SES	-0.09 (-0.16 ; -0.02)	-0.28 (-0.35 ; -0.21)
Road traffic noise	0.24 (0.07 ; 0.41)	0.18 (0.02 ; 0.33)
Neighborhood SES* road traffic noise	-0.02 (-0.08 ; 0.03)	-0.001 (-0.06 ; 0.06)
*B-N variance*	*0.76 (0.73 ; 0.79)*	*0.77 (0.74; 0.80)*
In the residential neighborhood (N = 6539)		
Neighborhood SES	-0.14 (-0.21 ; -0.07)	-0.31 (-0.38 ; -0.24)
Road traffic noise	0.11 (-0.08 ; 0.30)	0.10 (-0.08 ; 0.28)
Neighborhood SES* road traffic noise	0.09 (0.02 ; 0.17)	0.10 (0.02 ; 0.17)
*B-N variance*	0.77 (0.74 ; 0.80)	*0.79 (0.76 ; 0.82)*

Multilevel logistic regression models were estimated after excluding individuals with missing values for the two explanatory variables. Noise variable were continuous and standardized (Lden indicator). These variables were estimated at the place of residence or in the residential neighborhood that corresponded to the 75th percentile of noise values in each 500 m radius street network buffer around the place of residence. Neighborhood income and education were coded as 4-category (low, mid-low, mid-high, and high) ordinal variables. Abbreviation: SES: socioeconomic status; B-N: between neighborhood.

## Results and discussion

### Road traffic noise by neighborhood contexts

Figures 1 and 2 report the spatial distribution of outdoor transportation noise. Table 1 and Additional file 1: Table S2 provide levels of noise according to the neighborhood variables and according to the administrative division at the county level. When considering noise at the place of residence or the 25th and 50th percentiles of noise values in neighborhood buffers, outdoor road traffic noise surprisingly increased from Paris to the outer suburbs. However, when considering the 75th percentile, outdoor road traffic noise increased from the outer suburbs to Paris (Table 1). Regarding neighborhood education, for most noise variables, noise levels tended to increase from high educated neighborhoods to low educated neighborhoods. This finding is in contrast with a previous study of our group [23] that showed a positive relationship between neighborhood socioeconomic status, including neighborhood education, and exposure to noise. The discrepancy in the findings may be due to the fact that the previous study only considered the city of Paris, while the present work takes into account a broader territory from the Ile-de-France region. These updated findings are of interest for the assessment of situations of environmental injustice and highlight the need to study the variations in the patterns of environmental inequalities across various economic, social and cultural settings [28].

### Individual/neighborhood variables associated with annoyance due to road traffic

As also documented in previous literature [29-31], the annoyance due to road traffic was not associated with age or gender. The odds to report annoyance due to road traffic increased with decreasing household income, and independently with decreasing neighborhood income as well. After adjustment for the other individual/neighborhood factors, the odds of annoyance were higher in central urban neighborhoods than in urban and suburban neighborhoods, with a much higher prevalence of annoyance in central urban neighborhoods with an intermediate than with a high social standing (Table 2).

### Road traffic noise and annoyance due to road traffic

The analyses showed that outdoor road traffic noise was associated with annoyance due to road traffic, after adjusting for individual and neighborhood socioeconomic variables. In all the models tested (Tables 3 and 4), the risk of being annoyed by road traffic increased with the level of noise. For example, in models 2A and 2B (Table 3), the risk of being annoyed by road traffic was around 3 times higher (OR = 2.26; 95% CI: 1.58, 3.21 and OR = 3.07; 95% CI: 1.80, 5.25, respectively) for the participants in the highest class (70 – <80 dB(A)) than for those in lowest class (30 – <40 dB(A)) of road traffic noise. Other studies than ours have also assessed associations of annoyance with road traffic noise [32].

Comparing models 2A and 2B and comparing models 4A, 4B, 4C and 4D (Tables 3 and 4) suggest that it was not possible, due to the wide 95% confidence intervals (despite differences in OR), to conclude that stronger associations were observed when noise was assessed in the residential neighborhood than at the place of residence. A striking finding from the models assessing noise in the residential neighborhood was that a stronger relationship was documented with the 50th percentile of the noise level in the buffer than with the 25th percentile, and that the relationship was still stronger with the 75th percentile. A likely explanation is that the louder levels of noise captured by higher percentiles in the neighborhood have a particular impact on annoyance due to road traffic. However, it is important to note that road traffic noise measured at the place of residence and road traffic noise in the residential neighborhood (75th percentile) were independently associated with annoyance, when adjusted for each other.

As shown in Table 5, we documented interactions on the multiplicative scale between the effects on annoyance of road traffic noise estimated in the residential neighborhood or at the place of residence and of neighborhood income or neighborhood education [27]. These interactions indicated that the effect of road traffic noise in the residential neighborhood on annoyance due to road traffic was stronger in affluent and high educated neighborhoods than in deprived and low educated neighborhoods. This finding is coherent with our previous work demonstrating that the affluent part of Paris comprises particularly noisy roads [23]. These affluent and high educated neighborhoods were located in the central part of the Ile-de-France region, in Paris (Additional file 1: Table S1) which were also the noisiest neighborhoods when the 75th percentile of noise values in the buffer was taken into account (Table 1). The interaction was documented when noise levels in the residential neighborhood were assessed with the 75th percentile of noise values in the buffer, but not when they were assessed with the 25th or 50th percentiles. No interaction was documented between the effects of road traffic noise estimated at the place of residence and these neighborhood variables (Table 5). Also, no interaction was documented between the effect of any of the noise variables and individual socioeconomic variables (results not shown in a Table). Absolutely no interaction was documented on the additive scale (Additional file 1: Table S6).

### Rail traffic noise and annoyance due to road traffic

As opposed to outdoor road traffic noise, no associations were documented between outdoor rail traffic noise estimated in the residential neighborhood and annoyance related to road traffic (Additional file 1: Tables S3 and S4). Such an absence of relationship may be attributable, first to the fact that the survey question on annoyance was explicitly related to road traffic, and second to the fact that railway noise (because of the low density of the rail network) may be a weaker source of annoyance than road and aircraft noise [9]. Calculating the total length of railways and roads from data of the Institute of Urban Planning of the Ile-de-France region and of the National Geographic Institute, we found that the total length of the rail network represents only 2.1% of the total length of the railway and road network (cumulated) in the Ile-de-France region.

Contrary to rail traffic noise in the residential neighborhood, outdoor rail traffic noise estimated at the place of residence was associated with annoyance due to road traffic, with higher odds of annoyance in the highest class of noise (70 – <80 dB(A)) (Additional file 1: Table S3). After adjusting for road traffic noise at the place of residence, the association between rail traffic noise at the place of residence and annoyance due to road traffic also persisted. However, when modification of the relationship between road traffic noise at the place of residence and annoyance due to road traffic by rail traffic noise at the place of residence was tested, no interaction was documented (Additional file 1: Table S5). This absence of interaction suggests that we did not find support for our hypothesis that an alternative source of noise (rail traffic) may exacerbate the effects of road traffic noise on annoyance due to road traffic. No interactions between rail traffic noise and individual/neighborhood socioeconomic variables were also documented (results not shown in a Table).

### Between-neighborhood variance in annoyance due to road traffic

The variance between neighborhoods in the degree of annoyance was substantial in the empty models: equal to 0.75; 95% CI: 0.72, 0.78 in the samples for road traffic noise, and equal to 0.72; 95% CI: 0.69, 0.76 and 0.71; 95% CI: 0.67, 0.74 in the two samples for the analysis of rail traffic noise. As shown in Tables 3 and 4, and in Additional file 1: Tables S3 and S4, there was no evidence that the between-neighborhood decreased when individual/neighborhood variables and noise exposure were taken into account into the models. The fact that the between-neighborhood variance increased in some of the models when adding the covariates is due to the fact that coefficients in successive logistic models are not comparable to each other [33].

### Strengths and limitations

Strengths of this study include the large sample of participants with information available on a large spatial scale (the Ile-de-France region), the collection of data on the perception of road traffic for several thousands of participants, the fact that traffic noise from two important transportation modes was taken into account, the different types of measures of noise that were compared (at the place of residence and within neighborhood buffers, etc.) and the fact that the models were adjusted for multiple individual/neighborhood confounders.

A limitation of this study is the heterogeneity in the source and in the quality of the original transportation noise data provided by municipalities, inter-municipalities, and government services [34]. Another limitation includes the absence of *a priori* sampling in the recruitment of the participants, with differences in the probability of participation according to neighborhood profiles [35]. However, it is not clear whether and how annoyance due to road traffic might influence participation in the study. Finally, annoyance due to road traffic in the neighborhood was assessed with a single survey item from the RECORD questionnaire. This survey question did not allow us to distinguish between the different sources of road traffic nuisances (noise, smell, risk of injury), and could not be used to isolate annoyance related to road traffic noise. However, a study dealing with road traffic nuisances in the United Kingdom showed that with smokes and odors, road traffic noise was the main source of annoyance at the place of residence and when walking in the residential neighborhood [36]. Noise was also the first source of nuisance in the Ile-de-France region in the Health Barometer Study [1]. Besides, around 13% of people (49% of 26.3% (n = 1878) of people annoyed by noise at home) declared to be annoyed by road traffic noise at home in the Ile-de-France region in this study [1]. In the Health Barometer, the percentage of people annoyed by noise at home varied in a substantial way according to the location in the metropolitan area: 45.4% of people living in Paris are annoyed by noise at home vs. 29% of the residents of inner suburbs, and 25.6% of those who live in outer suburbs. The prevalence of people annoyed by road traffic noise found in the Health Barometer is relatively coherent with the prevalence of people annoyed by road traffic nuisances in our study, ie.,17.4% (n = 7290).Similarly, this percentage in our study showed variations between Paris (23.0%), the inner suburb (15.7%), and the outer suburb (15.5%).

## Conclusion and perspectives

Based on our large sample from a broad territory in the Ile-de-France region, disparities in exposure to road traffic noise were identified according to the educational level of the residents, with higher levels of exposure in low education neighborhoods. Such disparities were documented when noise was assessed at the place of residence and in the residential neighborhood. However, an inversion in the educational gradient of exposure was observed at the highest percentiles of noise exposure in neighborhood buffers (75th percentile), even if the differences between the educational groups were very small. Such patterns may be attributable to the fact that residents of low educated neighborhoods are exposed to a higher level of noise in most part of their neighborhood due to high-traffic and noisy highways, with the resulting noise reaching to a certain extent the places of residence and residential neighborhoods because of a weaker density of the urban network in outer suburbs. On the opposite, there may be a number of high educated neighborhoods with an intermediate level of noise exposure but that comprise very high traffic and noisy roads which the highest noise levels were more often included in residential neighborhoods due to a very dense urban network in the central part of the Ile-de-France region.

Associations were documented between road traffic noise and annoyance due to road traffic, after adjustment for individual/neighborhood factors. The association between rail traffic noise and annoyance due to road traffic was weaker. There was no strong evidence that the association was of different magnitude when noise was measured at the place of residence or in the residential neighborhood. However, the strength of the association between noise in the neighborhood and annoyance tended to increase when considering a higher percentile in the distribution of noise in each neighborhood. Additional analyses not reported here suggest that, once the 75th percentile of road traffic noise in the neighborhood was included into the model, there were no additional relationships with the 25th and 50th percentiles. What matters in the neighborhood as a determinant of annoyance is therefore the highest levels of noise exposure in the environment (as captured by the 75th percentile of noise level in the buffer), rather than the lower levels of noise in the remaining of the neighborhood (as captured by percentiles below the 75th). However, it is important to note that road traffic noise measured at the place of residence and road traffic noise in the residential neighborhood (75th percentile) were independently associated with annoyance, when adjusted for each other.

Interactions of effects indicated that the relationship between road traffic noise exposure in the residential neighborhood (75th percentile) and annoyance due to road traffic was stronger in the most affluent and high educated neighborhoods (i.e., those from Paris). This finding is surprising because authors usually hypothesize that low socioeconomic people are more sensitive to the effects of outdoor noise, e.g., because their dwellings are less correctly isolated [1,37]. However, first, it should be noted that other studies have hypothesized a higher sensitivity to noise among affluent people [17] based on the observation of a higher number of complaints in this population, which may be attributable to the fact that socially advantaged people have a higher awareness of and pay more attention to noise as an environmental exposure, in part because they feel they are able to avoid this exposure (e.g., by moving) contrary to low socioeconomic status people who might feel powerless to decrease their exposure level. Second, such an interaction may also be driven by the fact that a number of affluent and high educated neighborhoods are exposed to very high traffic roads in at least part of the neighborhood. For example, the assumption of a higher impact on annoyance of an increment in noise exposure at higher levels of the noise scale (e.g., higher impact of an increase from 65 to 75 than of an increase from 55 to 65 dB(A)) would explain the observed interaction. Third, there may be more behavioral explanations to this interaction. People living in the most affluent neighborhoods in urban centers with a large number of services are particularly engaged in walking in their neighborhood. Analyses of walking in the RECORD Study show that a high density of services and a high neighborhood educational level are independently associated with walking. Thus people residing in such neighborhood may be more exposed to road traffic noise in their neighborhood and particularly aware of it, which may also contribute to the reported interaction. Interestingly, the strongest associations between road traffic noise in the residential neighborhood and annoyance documented in affluent and high educated neighborhoods show that it is relevant to study the exposure to noise in the daily environments of people, such as their residential neighborhood.

Overall, our findings suggest that it is useful to take into account (i) the exposure to transportation noise during daily trips in the residential neighborhood rather than only the outdoor exposure level at the residence, (ii) different percentiles of noise exposure in the residential neighborhood, and (iii) the socioeconomic characteristics of the residential neighborhood to explain variations in annoyance due to road traffic in the neighborhood.

To better understand our findings and why there is a spatial discrepancy in the patterns of associations when noise exposure is assessed at the place of residence or in the residential neighborhood, new technologies of mobility and health assessment will be used in our future work. This project, funded by the French Agency for Food, Environmental and Occupational Health & Safety (ANSES) and by the French Environment and Energy Management Agency (ADEME), will combine Global Positioning System (GPS) tracking, assessment of individual noise exposure with noise sensors, and assessment of annoyance and health with ambulatory monitoring of health indicators. Such an approach will allow us to better characterize the complex interactions between the daily life environments, the multi-exposure to noise, and annoyance and health.

## Competing interests

The authors declare that they have no competing interests.

## Authors’ contributions

JM designed the specific study, conducted most of the analyses, and oversaw the drafting of the manuscript with BC (study coordinator). All authors have participated in the analysis and interpretation of the data, and in the review of the manuscript drafts. AH elaborated the neighborhood typology. NK participated in the analysis and interpretation of the data. FT also participated in the conception of the data collection protocol. All authors read and approved the final manuscript.

## Supplementary Material

Additional file 1: Table S1 Spatial distribution of neighborhood education and income, according to administrative division in counties (RECORD Cohort; N = 7290). **Table S2.** Spatial distribution of rail traffic noise, according to the administrative division in counties, neighborhood urban typology and neighborhood education (RECORD Cohort Study). **Table S3.** Associations estimated from multilevel logistic regression between rail traffic noise estimated at the place of residence (3A) and at the median noise value of 500 m radius street network buffers around the place of residence (3B) and annoyance due to road traffic, adjusted for individual/neighborhood socio-demographic factors (RECORD Cohort Study). **Table S4.** Associations estimated from multilevel logistic regression between rail traffic noise estimated at the place of residence (5A) and at the 25th (5B), 50th (5C), and 75th percentiles (5D) of noise values of 500 m radius street network buffers around the place of residence and annoyance due to road traffic, adjusted for individual/neighborhood socio-demographic factors (RECORD Cohort Study). **Table S5.** Modification of the association between road traffic noise at the place of residence and annoyance due to road traffic, by rail traffic noise at the place of residence, on the multiplicative and additive scales (RECORD Cohort Study; N = 3945). **Table S6.** Modification of the association between road traffic noise and annoyance due to road traffic, by neighborhood income and education, on the additive scale (RECORD Cohort Study). Click here for file
