# Supplementary material for: Transportation noise and annoyance related to road traffic in the French RECORD study
Source: Int J Health Geogr. 2013 Oct 2;12:44. doi: 10.1186/1476-072X-12-44 (PMC3850497; doi:10.1186/1476-072X-12-44)
Supplement: Additional file 1: Table S1 — Spatial distribution of neighborhood education and income, according to administrative division in counties (RECORD Cohort; N = 7290). Table S2. Spatial distribution of rail traffic noise, according to the administrative division in counties, neighborhood urban typology and neighborhood education (RECORD Cohort Study). Table S3. Associations estimated from multilevel logistic regression between rail traffic noise estimated at the place of residence (3A) and at the median noise value of 500 m radius street network buffers around the place of residence (3B) and annoyance due to road traffic, adjusted for individual/neighborhood socio-demographic factors (RECORD Cohort Study). Table S4. Associations estimated from multilevel logistic regression between rail traffic noise estimated at the place of residence (5A) and at the 25th (5B), 50th (5C), and 75th percentiles (5D) of noise values of 500 m radius street network buffers around the place of residence and annoyance due to road traffic, adjusted for individual/neighborhood socio-demographic factors (RECORD Cohort Study). Table S5. Modification of the association between road traffic noise at the place of residence and annoyance due to road traffic, by rail traffic noise at the place of residence, on the multiplicative and additive scales (RECORD Cohort Study; N = 3945). Table S6. Modification of the association between road traffic noise and annoyance due to road traffic, by neighborhood income and education, on the additive scale (RECORD Cohort Study). [file 1476-072X-12-44-S1.docx]

| **Table S1. Spatial distribution of neighborhood education and income, according to administrative division in counties (RECORD Cohort; N = 7290)** | | | | | | |
| --- | --- | --- | --- | --- | --- | --- |
| Variables | Neighborhood proportion  of highly educated residents (in %) | | | Neighborhood median income  (in Euros) | | |
|  | Mean | ±SD | Min/Max | Mean | ±SD | Min/Max |
| Ile-de-France region (N = 7290)  Outer suburbs  Inner suburbs  Paris | 33  40  51 | ±16  ±15  ±10 | [0 ; 75]  [0 ; 73]  [16 ; 69] | 24528  27619  28182 | 9218  10670  7280 | [0 ; 64520]  [0 ; 82098]  [12490 ; 66422] |
| P For Trend* | *36,43**  *<.0001* | | | *14,03**  *<.0001* | | |
| * P Values for trend were estimated from the Jonckheere-Terpstra test. All neighborhood variables were expressed as ordinal variables. Mean, Standard deviation, Minimum and Maximum were calculated, after excluding individuals with missing values for neighborhood variables. | | | | | | |

| **Table S2. Spatial distribution of rail traffic noise, according to the administrative division in counties, neighborhood urban typology and neighborhood education (RECORD Cohort Study)** | | | | | | | | |
| --- | --- | --- | --- | --- | --- | --- | --- | --- |
| Variables | Rail traffic noise at the place of residence (with the Lden indicator and in dB(A))  N = 3945 | | Rail traffic noise at the 25^th^ percentile of 500 m radius street network buffers  around the place of residence (with the Lden indicator and in dB(A))  N = 4265 | | Rail traffic noise at the median of 500 m radius street network buffers around the place of residence (with the Lden indicator and in dB(A))  N = 4265 | | Rail traffic noise  at the 75^th^ percentile of 500 m radius street network buffers around the place of residence (with the Lden indicator) in dB(A)  N = 4265 | |
|  | *Mean* | *±SD* | *Mean* | *±SD* | *Mean* | *±SD* | *Mean* | ±SD |
| Total | 36.02 | ±10.49 | 33.26 | ±6.10 | 35.16 | ±8.81 | 39.22 | ±12.79 |
| Ile-de-France region  Outer suburbs  Inner suburbs  Paris | 35.78  36.27  35 | ±12.07  ±8.29  ±0 | 32.37  34.16  34.14 | ±7.42  ±4.06  ±6.45 | 35.14  35.20  34.84 | ±10.72  ±6.25  ±7.78 | 40.19  38.38  35.05 | ±14.70  ±10.51  ±8.06 |
| P For Trend* |  | *27.16**  *<.0001* |  | *36.02**  *<.0001* |  | *26.13**  *<.0001* |  | *13.79**  *<.0001* |
| Neighborhood proportion of highly educated residents  Low  Mid-low  Mid-high  High | 36.50  35.99  36.10  34.68 | ±11.12  ±10.58  ±10.52  ±8.24 | 33.69  33.06  32.89  32.94 | ±6.93  ±6.28  ±4.86  ±4.51 | 35.92  35.04  34.22  34.50 | ±9.84  ±8.96  ±7.15  ±7.14 | 40.05  39.28  39.38  36.83 | ±13.49  ±12.83  ±13.05  ±10.07 |
| P For Trend* |  | *0.73**  *0.23* |  | *1.23**  *0.11* |  | *0.0035**  *0.50* |  | *-1.85**  *0.0318* |
| Neighborhood typology  Type 1: suburban, low social standing  Type 2: suburban, high social standing  Type 3: urban, low social standing  Type 4: urban, high social standing  Type 5: central urban, high social standing  Type 6: central urban, interm. social standing | 36.35  36.14  35.57  35.90  -  35.38 | ±12.29  ±11.69  ±8.53  ±9.16  -  ±5.67 | 33.12  32.93  33.51  33.39  30.50  34.45 | ±8.17  ±7.51  ±4.66  ±3.74  ±1.58  ±1.57 | 36.00  35.48  34.77  34.64  30.50  34.78 | ±11.44  ±10.44  ±7.13  ±6.28  ±1.58  ±3.07 | 40.65  39.64  38.42  38.69  30.50  36.81 | ±14.79  ±14.09  ±11.24  ±11.39  ±1.58  ±6.97 |
| P For Trend** |  | *253.62***  *<.0001* |  | *753.69***  *<.0001* |  | *275.41***  *<.0001* |  | *113.07***  *<.0001* |
| * P Values for trend were estimated from the Jonckheere-Terpstra test. ** P Values for trend were estimated from the Kruskall-Wallis test. All neighborhood variables were expressed as ordinal variables. Means and standard deviations were calculated, after excluding individuals with missing values for traffic noise and neighborhood variables. In the Ile-de-France region, “Paris” is the district 75; “inner suburbs” and “outer suburbs” gather respectively districts 92, 93, and 94 and districts 77, 78, 91, and 95. | | | | | | | | |

| Table S3. Associations estimated from multilevel logistic regression between rail traffic noise estimated at the place of residence (3A) and at the median noise value of 500 m radius street network buffers around the place of residence (3B) and annoyance due to road traffic, adjusted for individual / neighborhood socio-demographic factors (RECORD Cohort Study) | | | | |
| --- | --- | --- | --- | --- |
|  | Model 3A  N = 3945 | | Model 3B  N = 4265 | |
|  | OR | 95% CI | OR | 95% CI |
| Rail traffic noise estimated  (3A): at the place of residence  (3B): in the residential neighborhood  (Lden indicator)  (vs [ 30 – 40 dB(A) [ )  [ 50 – 60 dB(A) [  [ 60 – 70 dB(A) [  [ 70 – 80 dB(A) [ | 1.44  0.97  2.07 | (1.04 ; 2.00)  (0.66 ; 1.42)  (1.23 ; 3.50) | 1.15  1.02  3.24 | (0.83 ; 1.59)  (0.67 ; 1.57)  (0.94 ; 11.14) |
| *Between-neighborhood variance* | *0.75 (0.72 ; 0.79)* | | *0.75 (0.71 ; 0.79)* | |
| Multilevel logistic regression models were estimated after excluding individuals with missing values for rail traffic noise variables. These models were estimated between categorical noise variables and annoyance due to road traffic, adjusted for individual/neighborhood factors of basic model 1 (Table 2). Rail traffic noise was estimated at the place of residence in Model 3A and as the median value of 500 m radius street network buffers around the place of residence in model 3B. | | | | |

| Table S4. Associations estimated from multilevel logistic regression between rail traffic noise estimated at the place of residence (5A) and at the 25^th^ (5B), 50^th^ (5C), and 75^th^ percentiles (5D) of noise values of 500 m radius street network buffers around the place of residence and annoyance due to road traffic, adjusted for individual / neighborhood socio-demographic factors (RECORD Cohort Study) | | | | | |
| --- | --- | --- | --- | --- | --- |
| Rail traffic noise  at the place of residence | Rail traffic noise  in the residential neighborhood  (25^th^ percentile of  noise values of buffers) | Rail traffic noise  in the residential neighborhood  (median of noise values of buffers) | Rail traffic noise  in residential the neighborhood  (75^th^ percentile of  noise values of buffers) |  |  |
| OR (95% CI) | OR (95% CI) | OR (95% CI) | OR (95% CI) |  |  |
| Model 5A (N=3945) | Model 5B (N=4265) | Model 5C (N=4265) | Model 5D (N=4265) |  |  |
| 1.11 (1.02 ; 1.21) | 1.07 (0.99 ; 1.17) | 1.05 (0.96 ; 1.15) | 1.00 (1.91 ; 1.09) |  |  |
| *B-N variance: 0.75 (0.72;0.79)*  *Akaike: 19356.7* | *B-N variance: 0.75 (0.71;0.78)*  *Akaike: 20824.5* | *B-N variance: 0.75 (0.71;0.79)*  *Akaike: 20821.4* | *B-N variance: 0.75 (0.72;0.79)*  *Akaike: 20820.3* |  |  |
| Multilevel logistic regression models were estimated after excluding individuals with missing values for rail traffic noise variables. These models were estimated between standardized continuous noise variables and annoyance due to road traffic, adjusted for individual/neighborhood factors of basic model 1 (Table 2). Rail traffic noise was estimated in 500 m radius street network buffers around the place of residence; B-N: between-neighborhood. | | | | | |

| **Table S5. Modification of the association between road traffic noise at the place of residence and annoyance due to road traffic, by rail traffic noise at the place of residence, on the multiplicative and additive scales (RECORD Cohort Study; N=3945)** | |
| --- | --- |
| Variables | Annoyance due to road traffic |
|  | β (95% CI) |
| **On the multiplicative scale** |  |
| Road traffic noise | 0.25 (0.16 ; 0.34) |
| Rail traffic noise | 0.1 (0.01 ; 0.18) |
| Road traffic noise * rail traffic noise | -0.07 (-0.15 ; 0.01) |
| *Between-neighborhood variance* | *0.73 (0.70; 0.77)* |
| **On the additive scale** |  |
| Road traffic noise | 0.03 (0.02 ; 0.04) |
| Rail traffic noise | 0.01 (-0.01 ; 0.02) |
| Road traffic noise * rail traffic noise | -0.01 (-0.02 ; 0.01) |
| *Between-neighborhood variance* | *0.12 (-0.11 ; 0.13)* |
| Multilevel logistic regression models were estimated after excluding individuals with missing values for the two explanatory variables. The noise variables were continuous and standardized (Lden indicator). These variables were estimated at the place of residence. Annoyance due to road traffic was coded as a binary variable. | |

| Table S6. Modification of the association between road traffic noise and annoyance due to road traffic, by neighborhood income and education, on the additive scale (RECORD Cohort Study) | | |
| --- | --- | --- |
|  | Neighborhood proportion  of highly educated residents | Neighborhood median income |
|  | β - 95% CI | β - 95% CI |
| At the place of residence (N=6194) |  |  |
| Neighborhood SES | -0.01 (-0.02 ; -0.004) | -0.04 (-0.05 ; -0.03) |
| Road traffic noise | +0.04 (+0.01 ; +0.06) | +0.04 (+0.01 ; +0.06) |
| Neighborhood SES* road traffic noise | -0.01 (-0.01 ; +0.003) | -0.005 (-0.01 ; +0.004) |
| *Between-neighborhood variance* | *0.134 (0.129 ; 0.140)* | *0.134 (0.129 ; 0.140)* |
| In the residential neighborhood (N=6539) |  |  |
| Neighborhood SES | -0.02 (-0.03 ; -0.01) | -0.04 (-0.05 ; -0.03) |
| Road traffic noise | +0.03 (+0.001 ; +0.05) | +0.04 (+0.01 ; +0.06) |
| Neighborhood SES* road traffic noise | +0.01 (-0.004 ; +0.01) | +0.001 (-0.01 ; +0.01) |
| *Between-neighborhood variance* | *0.135 (0.130 ; 0.140)* | *0.135 (0.130 ; 0.140)* |
| Multilevel logistic regression models were estimated after excluding individuals with missing values for the two explanatory variables. Noise variable were continuous and standardized (Lden indicator). These variables were estimated at the place of residence or in the residential neighborhood that corresponded to the 75^th^ percentile of noise values in each 500 m radius street network buffer around the place of residence. Neighborhood income and education were coded as 4-category (low, mid-low, mid-high, and high) ordinal variables. Abbreviation: SES: socioeconomic status. | | |
